# The complete chloroplast genome of an aromatic Chinese pepper (*Zanthoxylum simulans*)

**DOI:** 10.1080/23802359.2017.1419082

**Published:** 2017-12-20

**Authors:** Na Hou, Gang Wang, Shi Jing Feng, An Zhi Wei

**Affiliations:** a College of Forestry, Northwest A&F University, Yangling, Shaanxi, China;; b Guizhou Academy of Forestry, Guizhou, China

**Keywords:** *Zanthoxylum simulans*, plastid genome, Chinese prickly-ash, germplasm resources conservation

## Abstract

The Chinese pepper (*Zanthoxylum simulans*) is a flowering plant in the family Rutaceae, native to eastern China and Taiwan. Like many other members of the Rutaceae, it is an economically important aromatic crop known for its volatile oil and nutrition. We determined the complete chloroplast genome sequences for *Z. simulans* using IIumina sequencing. The *Z. simulans* chloroplast has a total length of 158,461 bp; it consists of a large single copy (LSC) region of 85,568 bp, a small single copy region (SSC) length of 17,603 bp, and an inverted region (IR) of 27,645 bp. The genome encodes 132 annotated genes, including 87 protein-coding genes, 37 tRNA, and 8 rRNA. The overall GC content of the *Z. simulans* chloroplast genome was 38.5%. A phylogenomic analysis revealed that *Z. simulans* are clustered with *Z. bungeanum* within the genus *Zanthoxylum*.

The *Zanthoxylum simulans* is an angiospermae plant in the family Rutaceae, endemic to eastern China and Taiwan (Feng et al. 2016). It is one of several species of *Zanthoxylum* from which Sichuan pepper is produced, which is a commonly used spice in Desi Chinese cuisine, Chinese, Korea, Tibetan, Nepali, and Indian cuisine (Paik et al. 2005). The husk or hull (pericarp) around the seeds may be used whole, especially in Sichuan cuisine or Szechwan cuisine or Szechuan cuisine, and the finely ground powder is one of the ingredients for five-spice powder (Lee et al. 2016). The genus *Zanthoxylum* includes more than 45 species and 13 varieties in China (Feng et al. 2016).

Here, we assembled the complete chloroplast genome sequence of *Z. simulans* using the high-throughput Illumina paired-end sequencing data. The annotated plastid genome has been deposited into GenBank with the accession number: MF716524. Total genomic DNA was extracted from a single individual *Z. simulans* growing in Jiuzhou town Xianren Ba village, Guizhou, China (106°08′E, 26°16′N). DNA sample and voucher specimen of *Z. simulans* were deposited in the Botany Laboratory, Guizhou Academy of Forestry (Guiyang, Guizhou, China). After trimming the sequences, high-quality PE reads were aligned to the *Z. bungeanum* (NCBI Accession number: KX497031) (Liu and Wei 2017) using bowtie (Langmead and Salzberg 2012), and assembled into a genome using SPAdes assembler (Bankevich et al. 2012). Annotation was performed with Dual Organellar Genome Annotator (DOGMA) software (Wyman et al. 2004). We corrected the annotation with Geneious (Kearse et al. 2012).

The *Z. simulans* whole chloroplast genome is 158,461 bp in length. GC content was 38.5%. It contained large single copy (LSC) of 85,568 bp, a small single copy region (SSC) of 17,603 bp, and a pair of inverted repeat (IRs) of 27,645 bp. The genome encodes 132 functional genes, including 87 protein-coding genes, 37 tRNA, and 8 rRNA. The newly sequenced plastid genome is quite similar to other *Zanthoxylum* species in terms of overall organization, gene/intron content, gene order, and GC content (Lee et al. 2016; Liu and Wei 2017).

We performed a phylogenetic analysis based on the complete chloroplast genome of *Z. simulans* and six other species from the family Rutaceae, including three from the genus *Zanthoxylum* and three from the genus *Citrus*. *Leitneria floridana* (Simaroubaceae) was used as an outgroup. The Maximum Likelihood (ML) phylogenetic tree analysis was performed using RAxML (Stamatakis 2014). The phylogenetic tree showed that *Z. simulans* was most closely related to *Z. bungeanum* (Figure 1). The newly characterized *Z. simulans* chloroplast genome can be used for studies of the evolutionary history of *Zanthoxylum* and to investigate genetic subdivision within the remaining *Z. simulans* germplasm resources.

**Figure 1.**
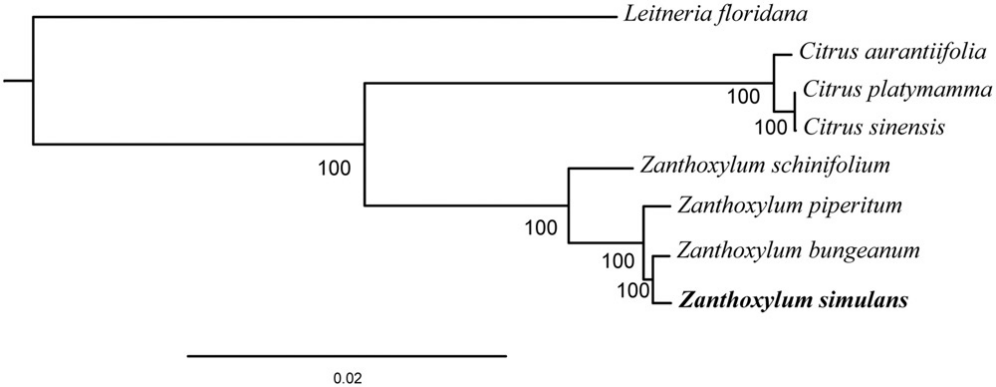
Maximum Likelihood (ML) phylogenetic tree based on complete chloroplast genome sequences of six species from the family Rutaceae using *L. floridana* of Simaroubaceae as an outgroup. Numbers on branches are bootstrap support values based on 10,000 iterations. All eight species’s accession numbers are listed as below: *Z. piperitum* KT153018, *Z. piperitum* NC_027939, *Z. schinifolium* KT321318, *C. aurantiifolia* KJ865401, *C. platymamma* KR259987, *C. sinensis* NC_008334, and *L. floridana* NC_030482.
